# An acquisition account of genomic islands based on genome signature comparisons

**DOI:** 10.1186/1471-2164-6-163

**Published:** 2005-11-18

**Authors:** MWJ van Passel, A Bart, HH Thygesen, ACM Luyf, AHC van Kampen, A van der Ende

**Affiliations:** 1 Academic Medical Center, Department of Medical Microbiology, Amsterdam, the Netherlands; 2 Academic Medical Center, Clinical Epidemiology and Biostatistics, Amsterdam, the Netherlands; 3 Bioinformatics Laboratory, Amsterdam, the Netherlands

## Abstract

**Background:**

Recent analyses of prokaryotic genome sequences have demonstrated the important force horizontal gene transfer constitutes in genome evolution. Horizontally acquired sequences are detectable by, among others, their dinucleotide composition (genome signature) dissimilarity with the host genome. Genomic islands (GIs) comprise important and interesting horizontally transferred sequences, but information about acquisition events or relatedness between GIs is scarce. In *Vibrio vulnificus *CMCP6, 10 and 11 GIs have previously been identified in the sequenced chromosomes I and II, respectively. We assessed the compositional similarity and putative acquisition account of these GIs using the genome signature. For this analysis we developed a new algorithm, available as a web application.

**Results:**

Of 21 GIs, VvI-1 and VvI-10 of chromosome I have similar genome signatures, and while artificially divided due to a linear annotation, they are adjacent on the circular chromosome and therefore comprise one GI. Similarly, GIs VvI-3 and VvI-4 of chromosome I together with the region between these two islands are compositionally similar, suggesting that they form one GI (making a total of 19 GIs in chromosome I + chromosome II). Cluster analysis assigned the 19 GIs to 11 different branches above our conservative threshold. This suggests a limited number of compositionally similar donors or intragenomic dispersion of ancestral acquisitions. Furthermore, 2 GIs of chromosome II cluster with chromosome I, while none of the 19 GIs group with chromosome II, suggesting an unidirectional dispersal of large anomalous gene clusters from chromosome I to chromosome II.

**Conclusion:**

From the results, we infer 10 compositionally dissimilar donors for 19 GIs in the *V. vulnificus *CMCP6 genome, including chromosome I donating to chromosome II. This suggests multiple transfer events from individual donor types or from donors with similar genome signatures. Applied to other prokaryotes, this approach may elucidate the acquisition account in their genome sequences, and facilitate donor identification of GIs.

## Background

From the many different prokaryotic genomes that have been sequenced in the last decade, it has been concluded that horizontal gene transfer (HGT) contributed significantly to the shape and size of microbial genomes [1]. Initially, HGT was regarded as an oddity in microbial genetics, with only a few antibiotic resistance genes in circulation. Currently, the estimates of putatively horizontally acquired DNA range from 0.5% in the endocellular symbiont *Buchnera *sp. APS genome up to 25% in the euryarchaeal *Methanosarcina acetivorans *genome, with an average of 14% in 116 prokaryotic genomes [2].

This genomic patchwork is clearly visible in the amount of genomic islands (GIs) detected in microbial genomes [3]. As more genomes of environmental strains are being sequenced, a variety of GIs providing diverse metabolic capacities are discovered in these non-pathogenic strains, emphasising that lateral genetic transfer is not limited to virulence traits [4]. Previous work on the functional categories of putative transferred genes resulted in the complexity theory, which contemplates the negative correlation between the probability of efficient acquisition and participation of the transferred genes in complex interactions [5]. Recently, it was claimed that transferred genes are biased towards functional categories associated with the cell surface, pathogenicity and DNA binding genes, although the proportion of putative genes with unknown functions remains very high in acquired sequences [2]. Dobrindt and co-workers explain acquisition efficiency mainly in terms of fitness increase [4]. These findings imply that diverse and interesting capacities are being exchanged between micro organisms.

In addition to their occasional location between mobile elements, such as phage sequences and insertion sequences suggesting a heterologous origin [6-8], GIs have been found to be compositionally different from their host with regard to codon usage and GC content as well as dinucleotide composition. Both chromosomes of *V. vulnificus *CMCP6 contain a large number of GIs as identified by Garcia-Vallve and co-workers. In their Horizontal Gene Transfer Database (HGT-DB) 10 and 11 large (>10 kbp) putatively horizontally acquired gene clusters are described for *V. vulnificus *CMCP6 chromosome I and chromosome II, respectively. These putative GIs have been identified parametrically using the GC-content, the codon usage and the amino acid usage [9].

In this study, we assessed the relatedness and acquisition account of GIs present on chromosomes I and II of *V. vulnificus *CMCP6, using the genome signature as a measure of similarity between these islands. For this analysis we modified our previously described application δρ-web, which allows dinucleotide composition dissimilarity comparisons between an input sequence and a representative genome sequence [10,11]. The newly developed algorithm, Compare_Islands, allows comparisons between the genome signatures of GIs with each other and that of a selectable genome sequence, and enables a sequence composition similarity grouping, in which the δ* value variations of the different fragments are adjusted for sequence length.

## Results

### Developing the algorithm Compare_ Islands

A web based application calculating the genomic dissimilarity values between diverse input sequences is offered at our website also featuring δρ-web ([10],. Genomic islands (with a sequence length of 10 kbp and up [12]) may be compared in this application against other large input sequences. The output comprises a matrix with the number of input sequences and the genome sequence with which the user compares the GIs (in our case *V. vulnificus *chromosome I). The δ* values between these islands are subsequently adjusted for size-dependent signature variation. For these settings a hierarchic clustering is carried out in R.

### Calculating the genomic dissimilarity between islands on chromosome I of *V. vulnificus *CMCP6

**Figure 1:**
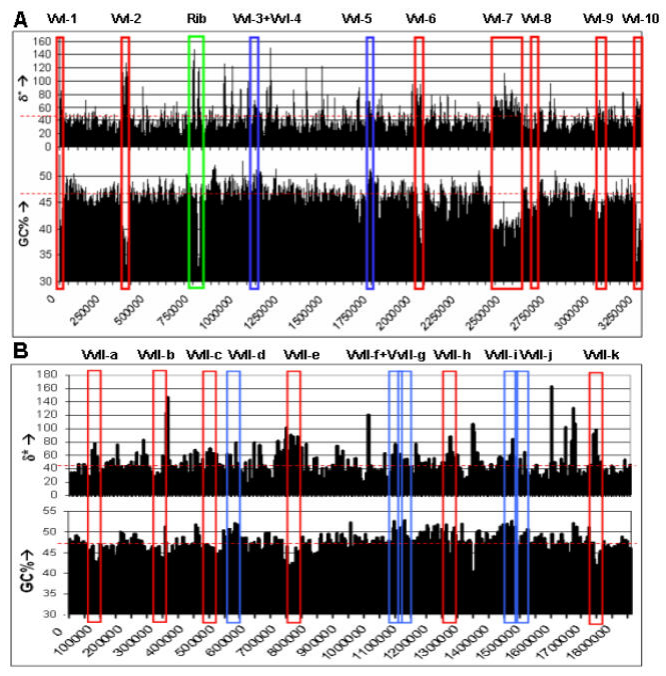
Overview of the two *V. vulnificus *chromosomes. Schematic representation of the δ* values and GC content of large putative horizontally transferred gene clusters in A) chromosome I and B) chromosome II of *V. vulnificus *CMCP6 using a window size of 5 kbp (x-axis represents chromosome position). Red depicts the low GC content GIs, while blue depicts the high GC content GIs. In green, a large ribosomal protein gene cluster is depicted (Rib). The horizontal dashed red line represents the average δ* value and GC percentage, respectively.

Initially, we assessed the composition dissimilarities among the 10 chromosome I GIs and the *Vibrio *chromosome I (table 1, Fig. 1A). Except for VvI-8, all of these GIs display a high genomic dissimilarity with chromosome I of *V. vulnificus*. Cross-referring these islands with the alternative GI detection tool IslandPath [13] verifies that VvI-8 is not considered anomalous in GC percentage or dinucleotide composition, but is dissimilar in codon usage. Anomalous loci VvI-3, VvI-4, and VvI-5 display a high GC percentage compared to the genomic values, whereas all other GIs have a lower GC percentage (table 1, Fig. 1A).

**Table 1:** The specifics of all large putative horizontally acquired gene clusters from chromosome I of *V. vulnificus *CMCP6, including their δ* (against *V. vulnificus *chromosome I) and GC composition values compared with chromosome I of *V. vulnificus *CMCP6. Vv5%, Vv10% and Vv10% are non-anomalous genomic fragments used as a reference clade. Ct1 and Ct2 represent *Chlamydia trachomatis *genomic fragments and are used as outgroups. The three *V. vulnificus *CMCP6 islands identified by Zhang and Zhang [22] are included in the last column. The presence of the *V. vulnificus *CMCP6 putative GIs in *V. vulnificus *YJ016 is verified by Web-ACT analysis [15].

**Locus**	**Coordinates**	**Size (bp)**	**Presence in *V. vulnificus *YJ016 chromosome I (fraction bp similar)**	**GI characteristics**	δ* (× 1000)	genomic fragments with a lower δ*	**GC%**	**genomic fragments with a lower GC%**	**Islands identified by [22].**
**VvI-1**	6115–17532	11417	Partially present (3': 2325/11417)	Preceeded by a transposase (VV10005)	59.4	89.6%	41.3%	5.57%	
**VvI-2**	355728–393737	38010	Partially present (3': 2343/38010)	Bordered by phage integrase (VV10372)	87.0	100%	37.8%	1.16%	VVGI-2
**VvI-3**	1094281–1109572	15292	Entirely present	-	42.7	81.3%	49.7%	95.3%	
**inter**	1109572–1122005	12433	Entirely present	-	48.4	82.9%	49.6%	95.1%	
**VvI-4**	1122005–1138423	16419	Entirely present	-	40.4	80.9%	50.7%	99.5%	
**VvI-5**	1749663–1764864	15202	Entirely present	-	46.9	85.6%	50.5%	98.6%	
**VvI-6**	2017768–2042744	24976	Partially present(3': 50/24976)	Putative integrase (VV12048)	74.4	98.5%	40.3%	2.29%	
**VvI-7**	2437730–2603335	165606	Highly dispersed	Superintegron integrase (VV12401), plasmid stabilisation protein encoding genes (VV12410))	57.8	100%	41.1%	5.26%	VVGI-1
**VvI-8**	2649661–2664017	14357	Entirely present	-	27.0	41.7%	42.8%	8.33%	
**VvI-9**	3033569–3043967	10399	Largely present (3': 6834/10399)	Preceded by a transposase (VV12969)	51.5	82.5%	42.1%	7.94%	
**VvI-10**	3260213–3279905	19692	Largely absent (few limited blocks of identity)	Putative transposase (VV13182)	64.6	95.8%	39.8%	1.81%	VVG1-3
**VvI-7a**	2437730–2519809	82080	Similar as VvI-7	See VVI-7	62.4	100%	40.8%	2.56%	Similar as VvI-7
**VvI-7b**	2519810–2603335	83526	Similar as VvI-7	See VVI-7	53.1	100%	41.4%	2.56%	Similar as VvI-7
**VvI-5%**	1155000–1170000	15001	Entirely present	-	14.0	6.0%	47.3%	59.6%	
**VvI-10%**	570000–585000	15001	Entirely present	-	17.2	10.6%	46.4%	54.1%	
**VvI-25%**	270000–285000	15001	Entirely present	-	21.2	24.3%	46.5%	38.5%	
**Ct1**	270000–285000^#^	15001	NR^&^	NR	167	NR	40%	NR	
**Ct2**	305000–320000	15001	NR	NR	169	NR	39%	NR	

ˆ) Coordinates from the *V. vulnificus *YJ016 chromosome I (note; the intergenic space beteen VVI0525-VV10526 in *V. vulnificus *YJ016 chromosome 1 was not included)

#) Coordinates for the *C. trachomatis *sequences are related to their respective genome sequence (Accession number AE001273).

^&^) NR denotes not relevant

**Figure 2:**
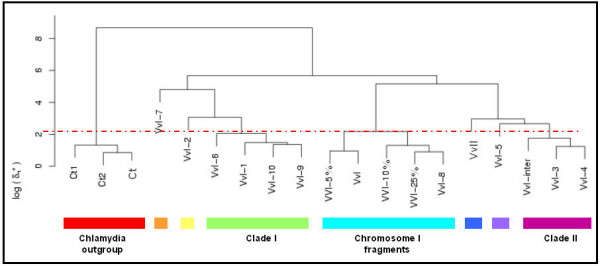
Hierarchic clustering with complete linkage of the *V. vulnificus *GIs (as described in table 1) based on the genome signature. Three non-anomalous genomic fragments (indicated with Vv5%, Vv10% and Vv25%) represent the conservative *V. vulnificus *(VvI) genomic variability, and this clade forms the cut-off value for the different clades (with the red dotted line; clades are indicated with black boxes). The *Chlamydia *clade consists of two genomic fragments (Ct1 and Ct2) and the genome sequence of *C. trachomatis*. VvII represents *V. vulnificus *chromosome II.

As the imprint of the global signature is locally pervasive on the scale ranging from 50 kbp down to 125 bp [14], compensating for the genomic dissimilarity variation allows us to adjust the genomic dissimilarity for different variations with sequence length among the 10 GIs (for unadjusted δ* values between the GIs from *V. vulnificus *CMCP6 chromosome I see additional file 1). A hierarchic clustering analysis was carried out with normalised δ* values to assess the compositional relatedness of the GIs (Fig. 2). As a reference clade of compositionally similar fragments, three 15 kbp fragments of regions outside the genomic islands of chromosome I with δ* values lower than that of 5%, 10% and 25% of all chromosomal fragments of 15 kbp, respectively, were included in this analysis to indicate clade cut-off values (see material and methods). In addition, two 15 kbp fragments (Ct1 and Ct2) as well as the complete genome sequence of *Chlamydia trachomatis *were included as out-groups (table 1).

The hierarchical clustering analysis showed that the two *C. trachomatis *fragments grouped together with the *C. trachomatis *genome sequence, and apart from all other fragments, as expected. VvI-8, with the lowest genomic dissimilarity compared to the genome sequence, grouped together with the 3 chromosomal fragments VvI-5%, VvI-10% and VvI-25%, and the chromosome I of *V. vulnificus *(VvI).

Using the branching point of the three non-anomalous reference fragments with the complete chromosome as a cut-off limit, two distinct clades are distinguished, formed by VvI-1, VvI-6, VvI-9 and VvI-10 (clade I) and by VvI-3 and VvI-4 (clade II), respectively. The remaining VvI-2, VvI-5 and VvI-7 are singleton GIs. The normalised δ* between VvI-3 and VvI-4 is very low, and together with the sequence proximity in the genome we suggest that these regions might actually be part of one larger anomalous gene cluster. Supportive to this notion is the very low composition dissimilarities between the region between these islands (labelled "VvI-inter" in the tables and figures) and GIs VvI-3 and VvI-4, all three fragments of comparable length (table 1). Hence, the three fragments group together in the hierarchic cluster analysis (Fig. 2).

VvI-1 groups together with VvI-6, VvI-9 and VvI-10. However, VvI-10 and VvI-1 are located at start and the end of the annotation of chromosome I. As this chromosome is circular, these islands are in fact adjacent, which can also be seen on the graphical output of IslandPath [13] at . The low δ* scores between these two regions suggest a similar dinucleotide composition and therefore these regions actually form one island.

We tested compositional similarity consistency by splitting the superintegron VvI-7 in two parts (VvI-7a and VvI-7b), after which the clustering analysis is repeated. The topology of the tree remains intact, and the two superintegron parts are clustered together below our threshold (see additional file 1).

Comparison of chromosome I of *V. vulnificus *CMCP6 with that of YJ016 by Web-ACT [15] showed that the reference sequences (Vv5%, Vv10% and Vv25%) of chromosome I of CMCP6 as well as the compositionally non-anomalous GI VvI-8 are all present in YJ016. In addition, all GIs comprising clade II are also present in YJ016 (table 1). In contrast, all clade I GIs (VvI-1, VvI-6, VvI-10 and (to a lesser extent) VvI-9) as well as VvI-2 and VvI-7 GIs are (largely) absent in YJ016. These results indicate that in contrast to *V. vulnificus *YJ016, *V. vulnificus *CMCP6 gained GIs belonging to one cluster, consistent with the notion of a single acquisition event of these GIs, or exclusive exposition to a specific donor.

### Calculating the genomic dissimilarity between GIs from both chromosomes of *V. vulnificus *CMCP6

Interestingly, the cluster analysis in figure 2 shows deep branching between chromosome I (VvI) and chromosome II (VvII), indicating substantial dissimilarity between these chromosomes. To assess the relationship between the GIs on chromosome I and on chromosome II we next included 11 putatively horizontally acquired gene clusters with length >10 kbp of chromosome II, as identified by the HGT-DB [9] in the analysis. First, the composition dissimilarities between the 11 chromosome II GIs and the *Vibrio *chromosome II was assessed. All GIs from chromosome II are anomalous in δ*, GC content or both (table 2, Fig. 1B).

**Table 2:** The specifics of all large putative horizontally acquired gene clusters from chromosome II of *V. vulnificus *CMCP6, including their δ* (against *V. vulnificus *chromosome I) and GC composition values compared with chromosome II of *V. vulnificus *CMCP6. Vv5%, Vv10% and Vv10% are non-anomalous genomic fragments used as a reference clade. Ct1 and Ct2 represent *Chlamydia trachomatis *genomic fragments and are used as outgroups. Zhang and Zhang [22] did not test V. vulnificus CMCP6 chromosome II.

**Locus**	**Coordinates**	**Size (bp)**	**Present in *V. vulnificus *YJ016 chromosome II (fraction bp similar)**	**GI characteristics**	δ* (× 1000)	Genomic fragments with a lower δ* %	**GC%**	**Genomic fragments with a lower GC%**
**VvII-a**	89575–104013	14438	Entirely present	tRNA synthetase	35.5	74.8%	41.8%	1.6%
**VvII-b**	302441–313330	10889	Entirely present	-	40.8	75.1%	42.5%	4.1%
**VvII-c**	452124–462927	10803	Largely present (7512/10803)	Transposase (VV20421)	56.9	94.1%	44.5%	11.8%
**VvII-d**	541308–554178	12870	Entirely present	-	61.7	96.5%	52.6%	100%
**VvII-e**	715669–749860	34191	Entirely absent	Transposases (VV20693 and VV20695)	71.6	100%	42.9%	1.9%
**VvII-f**	1064764–1077776	13012	Entirely present	-	54.6	93.6%	51.9%	100%
**VvII-g**	1083544–1106468	22924	Entirely present	-	38.1	87.5%	51.7%	100%
**VvII-h**	1227530–1239793	12263	Entirely present	-	44.8	82.7%	50.6%	96.0%
**VvII-i**	1420351–1433728	13377	Entirely present	-	47.6	90.5%	50.8%	96.4%
**VvII-j**	1446375–1462593	16218	Entirely present	-	60.2	95.6%	51.8%	100%
**VvII-k**	1724928–1739885	14957	Entirely present	-	62.0	95.1%	42.7%	4.9%
**VvII-5%**	105000–120000	15001	Entirely present	-	11.9	4.9%	46.1%	27.9%
**VvII-10%**	495000–510000	15001	Entirely present	-	15.1	10.7%	47.2%	52.5%
**VvII-25%**	960000–975000	15001	Entirely present	-	18.8	19.7%	48.1%	66.4%

**Figure 3:**
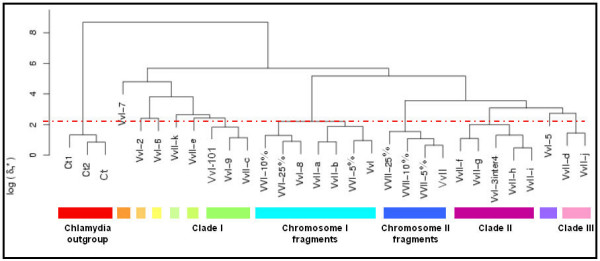
Hierarchic clustering with complete linkage of the *V. vulnificus *GIs from both chromosomes (as described in tables 1 and 2) based on the genome signature. For both chromosomes three non-anomalous genomic fragments are included, which represent the conservative *V. vulnificus *(VvI and VvII) genomic variability. VvI and VvII represent *V. vulnificus *chromosome I and II, respectively. VvI-3inter4 and VvI-101 represent the concatenated islands of VvI-3, VvI-inter and VvI-4 and of VvI10 and VvI-1 respectively.

Next, clustering analysis of the GIs of chromosome I and chromosome II was performed, in which the islands that were found to be compositionally very similar in figure 2 and in close proximity (VvI-1 and VvI-10, and VvI-3 and Vv-4), were taken as single entries, designated VvI-101 and VvI-3inter4, respectively. Addition of the GIs of chromosome II did not alter the overall topology of the clustering of the GIs of chromosome I (Fig. 3). As expected, the reference sequences group with the chromosomes from which they have been taken, while the Chlamydia chromosome and chromosomal fragments remain an outgroup. Cluster analysis yields 11 different branches above our conservative threshold for the 19 distinct GIs and the two chromosomes. We find that two GIs (VVII-a and VVII-b) from chromosome II are clustered with chromosome I, whereas one chromosome II GI (VvII-c) clusters with clade I and four chromosome II GIs (VvII-f, VvII-g, VvII-h and VvII-i) with clade II. In addition, two GIs (VvII-e and VvII-k) from the second chromosome are singletons, which branch just above our conservative threshold. Finally, the remaining two GIs (VvII-d and VvII-j) of chromosome II form a new clade III (Fig. 3).

## Discussion

Dinucleotide composition comparisons between different GIs may identify loci potentially originating from a compositionally similar donor. Identifying potential donors of acquired sequences facilitates the study of gene flow in the biosphere and the identification of the acquisition account may help understand how horizontal gene transfer influences genome evolution. We developed the algorithm, Compare_Islands, allowing comparisons between the genome signatures of GIs with each other and that of a selectable genome sequence, and enables a sequence composition similarity grouping. Robustness of the clustering methods was demonstrated by the clustering of two chromosomal fragments of *C. trachomatis *with its complete genome. In addition, normal chromosomal I and II fragments group with the complete chromosome I and chromosome II, respectively.

In the present study of *V. vulnificus *CMCP6, we found that some previously identified GIs are compositionally similar to each other, suggesting that they were derived from one donor or (compositionally) similar donors. For three clades (clade I clade II and clade III, Fig. 3), this implies either multiple transfer events from one donor or a single acquisition event followed by dispersion of the acquired fragment into multiple regions of the host genome thereafter. It should be noted however that a "clade" of two or more GIs does not necessarily imply evolutionary related donors of these GIs, as unrelated but compositionally similar donor types cannot be excluded.

There is some uncertainty as to what dissimilarity levels would be expected when comparing two islands, assuming that they come from donors with a similar genomic signature (ρ*), because the statistical fluctuations of ρ* can differ between candidate donors. In any event it is clear that some islands are substantially more similar to each other than they are to the host genome (e.g. VvI-3 and VvI-4) and could originate from closely related donors, while others (such as VvI-3 and VvI-6) are too different from each other to support the hypothesis of a recent common origin.

In this study we calculated the genomic dissimilarity scores of previously annotated putative GIs against *V. vulnificus *chromosome I, II and each other, whereas the chromosome contains many (more) acquired sequences with different signatures. Dissimilarity scores between the GIs and the genome would be more pronounced if the genome was purged of the acquired sequences. More pronounced dissimilarity scores would result in a more distinct cladification in the hierarchic clustering.

In addition, it is known that the discriminating ability of oligonucleotide composition comparisons is increased when longer motifs are used [16,17]. Therefore, if a higher resolution is considered necessary in order to compare different GIs, tetranucleotide or even longer oligonucleotide composition values may be of help. However, as the pervasive properties over large sequences has not been assessed per se for larger oligonucleotide motifs, the genome signature remains the most appropriate parameter for this sort of compositional analyses.

Of the *V. vulnificus *CMCP6 GIs, we propose that VvI-8 has not been horizontally transferred from a compositionally different donor, based on a low genomic dissimilarity with its respective host chromosome, a similar GC content compared to the chromosomal value and the absence of mobility elements (such as transposases or insertion elements) in or around this locus. Alternatively, VvI-8 might be acquired from a compositionally similar donor (e.g. a related Vibrio species) or it may have been acquired an evolutionary long time ago, resulting in a highly ameliorated fragment [18]. While high δ* values between GIs and the host genome sequence are indicative for acquisition from non-related donors via horizontal transfer, low δ* values cannot exclude recent acquisition events from compositionally similar donors, such as lateral gene transfer between related species [19]. It is of importance to note that parametric analyses can only indicate potential acquired regions by compositionally discordance. More elaborate strain analyses should subsequently provide further evidence of actual acquisition events, as has been done recently in *V. cholerae *and *V. Vulnificus *YJ016 with regard to GIs [20].

Various parameters have been described, such as the codon usage and the amino acid bias, that enable the identification of anomalous DNA in sequenced genomes (see for an extensive assessment [21]). Although improvements have been made in increasing the resolution obtained by individual parameters [14,22], a single parameter might not find all anomalous regions, and a combination of approaches obviously is preferred, as was already previously suggested [1]. An advantage of genome signature analyses is their applicability to identify anomalous DNA regions containing large stretches of noncoding DNA or small putative genes. In contrast, codon usage disregards the information in non-coding sequences and may not be feasible for very small open reading frames (<300 bp) such as ORFans [9].

From the GIs in *V. vulnificus *CMCP6, we propose that those in clade I, comprising the compositionally similar VvI-101, VvI-9 and VvII-c were acquired from one donor-species. This may either have been a single acquisition event followed by intra- and interchromosomal dispersal, or a series of acquisition events. VvI-10 and VvI-1, previously annotated as separate GIs, may be considered one GI for their actual proximity and compositional similarity (making a total of 19 GIs in *V. vulnificus *CMCP6). This emphasizes that linear analyses of circular genomes should be considered with care [22].

Similar to clade I, the GIs forming clade II, VvI-3 VvI-4, VvI-5, VvII-f, VvII-g, VvII-h and VvII-i may have been acquired successively from a compositionally similar donor or may have been dispersed upon a single acquisition event. Our results indicate that VvI-3 and VvI-4 are in fact part of one anomalous gene cluster, as the inter-island sequence displays a low dissimilarity with both VvI-3 and VvI-4, and a similarly ordered island is present in the related *V. vulnificus *YJ016 chromosome I. This single island has most likely been acquired in a single step, as recombination adjacent to islands originating from HGT events is considered unlikely [23,24].

Previously, the results of Chen and co-workers indicated that interchromosomal exchange had taken place between the two chromosomes in the various Vibrio genome sequences [25], and in *Vibrio cholerae *it was suggested that the second replicon itself may have been acquired horizontally [26]. Our results of a separate clustering of the chromosomes, as well as the clustering of GIs located on chromosome II with chromosome I support these findings. Figure 3 shows that the Vibrio chromosome I clade also contains GIs of chromosome II and it is appealing to speculate that VvII-a and VvII-b originally come from from chromosome I. In contrast, the Vibrio chromosome II clade does not contain any GIs of chromosome I or chromosome II, which may suggest that interchromosomal transfer of large anomalous gene clusters in *V. vulnificus *CMCP6 was unidirectional.

Concluding, our application Compare_Islands enables genome composition analyses with selectable window sizes and compositional comparisons between large sequences such as genomic islands. This allows an appraisal of the acquisition account of the large number of available prokaryotic genomes. In the case of *V. vulnificus *CMCP6, we propose a maximum number of 10 compositionally different donors for 19 distinct GIs. These results suggests that *V. vulnificus *accepted DNA from (compositionally) different sources, from some sources it accepted more DNA than from others, and a unidirectional flux of GIs from chromosome I to chromosome II is proposed.

## Methods

The strategy is based on the dinucleotide relative abundance values or genome signature (ρ*XY). As published previously by Karlin and Burge, each genome has its own typical dinucleotide relative abundance values, which are conserved between related species, as they are thought to result from the DNA repair and replication machinery [27]. Although the genome signature is found to be constant in 50 kbp windows [8], smaller windows can be used to identify anomalous sequences [28]. This is done by calculating the average dinucleotide relative abundance difference in a size dependent manner between the input sequence and a (closely related) representative genome sequence. This approach has been described previously and turned into a web application [10].

The comparisons between different large input sequences, such as GIs from the same strain, can be performed similarly. It may identify islands with similar genome signature values, indicating a compositionally similar donor or (dispersion effects of) the same acquisition event. On the other hand it may identify GIs with such different composition values that a compositionally similar donor is unlikely. The theory behind this approach is based on the results from Jernigan and Baran, which state that dinucleotide composition differences between sequences of different sizes can be calculated as the imprint of the global signature is locally pervasive on all scales [14]. Jernigan and Baran suggested, as a first approximation, that the dissimilarity between a genomic sequence and its host genome be corrected for the length of the sequence: log(delta*_norm) = log(delta*) -0.5 log(length). Empirical studies by Jernigan and Baran showed that the coefficient to log(length) for most organisms vary between -0.5 and -0.35. However, since the (hypothetical) donor organism is unknown and because the signature of the genomic islands could easily show a different statistical behaviour from that of genomic DNA, we will assume a coefficient of -0.5. Therefore, the normalized δ* is the raw δ* divided by the square root of the product of the inverted lengths of the two islands.

In order to set a conservative level of relatedness, we include 3 chromosomal fragments of 15 kbp with low δ* values compared to the complete chromosome; i.e. 3 different fragments with δ* values lower than that of 5%, 10% and 25% of all chromosomal fragments of 15 kbp, respectively. The highest branching point in the clustering of these three fragments compared to the genome sequence is considered a conservative cut-off value for a clade, as this clade indicates relatively related fragments. Hierarchic clustering is carried out in R [29].

The Compare_Islands application is available at  and includes user guidelines [11].

### Sequences

Ten and 11 large putatively horizontally acquired gene clusters with length between 10 kbp and 166 kbp from *V. vulnificus *CMCP6 chromosome I and chromosome II, respectively (as identified by Garcia-Valve and colleagues and presented in the Horizontal Gene Transfer Database (HGT-DB [9])) were obtained via the Position Search/Segment Retrieval tool [30] using the coordinates from the HGT-DB (for chromosome I, see table 1, for chromosome II see table 2). Acquired sequences shorter than 10 kbp were ignored in this analysis as GIs are described to vary between 10–200 kbp [7]. Three of these putative GIs on chromosome I correspond to three previously identified islands by Zhang and Zhang [22]; for example, the large VvI-7 (166 kbp, table 1), is similar to a large super integron identified in the related strain *V. vulnificus *YJ016 [25], albeit highly dispersed. The 10 GIs of chromosome I were numbered VvI-1 to VvI-10 according to their position in the annotation of the chromosome, while the 11 GIs of chromosome II were assigned by VvII-a to VvII-k.

## Authors' contributions

MWJvP, AB and AvdE devised the study and wrote the article, ACML and AHCvK set up the computational algorithms and HHT supplied the statistical background and calculations.

## Supplementary Material

Additional File 1 This table shows the genomic dissimilarity (δ*, × 1000) values between all GIs of chromosome I of *V. vulnificus *CMCP6. In green, all low δ* values are indicated, which are clustered together in figures 1 and 2. Click here for file
