# Supplementary material for: An acquisition account of genomic islands based on genome signature comparisons
Source: BMC Genomics. 2005 Nov 18;6:163. doi: 10.1186/1471-2164-6-163 (PMC1310630; doi:10.1186/1471-2164-6-163)
Supplement: Additional File 1 — This table shows the genomic dissimilarity (δ*, × 1000) values between all GIs of chromosome I of V. vulnificus CMCP6. In green, all low δ* values are indicated, which are clustered together in figures 1 and 2. [file 1471-2164-6-163-S1.doc]

Supplementary_Table_S1. Genomic dissimilarity scores (δ*) between all large putative horizontally acquired gene clusters in chromosome I of *V. vulnificus* CMCP6, as well as two *Chlamydia trachomatis* sequences (Ct1 and Ct2) and the complete genome of *C. trachomatis* (Ct) as outliers and 3 non-anomalous sequences from *V. vulnificus* chromosome I (VvI-5%, VvI-10% and VvI-25%). VvI denotes the complete chromosome I of *V. vulnificus* CMCP6. δ* values (1000x) of fragments which are clustered together in figure 1 are highlighted in green.

|  | **Length (bp)** | **VvI-1** | **VvI-2** | **VvI-3** | **inter** | **VvI-4** | **VvI-5** | **VvI-6** | **VvI-7** | **VvI-8** | **VvI-9** | **VvI-10** | **VvI** | **Vv5I-%** | **VvI-10%** | **VvI-25%** | **Ct1** | **Ct2** | **Ct** |
| --- | --- | --- | --- | --- | --- | --- | --- | --- | --- | --- | --- | --- | --- | --- | --- | --- | --- | --- | --- |
| **VvI-1** | 11417 | 0 | 58 | 93 | 103 | 95 | 97 | 36 | 60 | 53 | 30 | 27 | 59 | 59 | 66 | 61 | 118 | 122 | 110 |
| **VvI-2** | 38010 |  | 0 | 116 | 112 | 113 | 133 | 32 | 88 | 87 | 60 | 50 | 87 | 84 | 90 | 103 | 113 | 113 | 102 |
| **VvI-3** | 15292 |  |  | 0 | 33 | 23 | 36 | 114 | 67 | 65 | 93 | 100 | 43 | 41 | 53 | 50 | 207 | 208 | 198 |
| **inter** | 12433 |  |  |  | 0 | 31 | 55 | 114 | 70 | 64 | 97 | 108 | 48 | 54 | 54 | 60 | 208 | 208 | 198 |
| **VvI-4** | 16419 |  |  |  |  | 0 | 37 | 115 | 83 | 62 | 91 | 101 | 40 | 41 | 48 | 48 | 208 | 210 | 199 |
| **VvI-5** | 15202 |  |  |  |  |  | 0 | 116 | 87 | 52 | 86 | 103 | 47 | 48 | 43 | 42 | 209 | 210 | 200 |
| **VvI-6** | 24976 |  |  |  |  |  |  | 0 | 73 | 63 | 36 | 30 | 74 | 79 | 77 | 74 | 105 | 106 | 95 |
| **VvI-7** | 165606 |  |  |  |  |  |  |  | 0 | 57 | 58 | 70 | 58 | 61 | 66 | 58 | 164 | 160 | 152 |
| **VvI-8** | 14357 |  |  |  |  |  |  |  |  | 0 | 43 | 58 | 27 | 40 | 24 | 20 | 156 | 158 | 148 |
| **VvI-9** | 10399 |  |  |  |  |  |  |  |  |  | 0 | 26 | 52 | 59 | 52 | 49 | 135 | 139 | 127 |
| **VvI-10** | 19692 |  |  |  |  |  |  |  |  |  |  | 0 | 65 | 66 | 71 | 67 | 132 | 136 | 124 |
| **VvI** | 3281945 |  |  |  |  |  |  |  |  |  |  |  | 0 | 14 | 17 | 21 | 167 | 169 | 159 |
| **VvI-5%** | 15001 |  |  |  |  |  |  |  |  |  |  |  |  | 0 | 26 | 33 | 170 | 172 | 161 |
| **VvI-10%** | 15001 |  |  |  |  |  |  |  |  |  |  |  |  |  | 0 | 24 | 170 | 172 | 161 |
| **VvI-25%** | 15001 |  |  |  |  |  |  |  |  |  |  |  |  |  |  | 0 | 167 | 169 | 158 |
| **Ct1** | 15001 |  |  |  |  |  |  |  |  |  |  |  |  |  |  |  | 0 | 21 | 18 |
| **Ct2** | 15001 |  |  |  |  |  |  |  |  |  |  |  |  |  |  |  |  | 0 | 13 |
| **Ct** | 1042519 |  |  |  |  |  |  |  |  |  |  |  |  |  |  |  |  |  | 0 |
